# The artiodactyl *APOBEC3 *innate immune repertoire shows evidence for a multi-functional domain organization that existed in the ancestor of placental mammals

**DOI:** 10.1186/1471-2199-9-104

**Published:** 2008-11-18

**Authors:** Rebecca S LaRue, Stefán R Jónsson, Kevin AT Silverstein, Mathieu Lajoie, Denis Bertrand, Nadia El-Mabrouk, Isidro Hötzel, Valgerdur Andrésdóttir, Timothy PL Smith, Reuben S Harris

**Affiliations:** 1 Department of Biochemistry, Molecular Biology and Biophysics, Institute for Molecular Virology, Beckman Center for Genome Engineering, University of Minnesota, Minneapolis, Minnesota 55455, USA; 2 University of Iceland, Institute for Experimental Pathology, Keldur v/Vesturlandsveg, 112 Reykjavík, Iceland; 3 Masonic Cancer Center, Biostatistics and Bioinformatics Group, University of Minnesota, Minneapolis, Minnesota 55455, USA; 4 DIRO, Université de Montréal, Montréal, Quebec, H3C 3J7, Canada; 5 Department of Veterinary Microbiology and Pathology, Washington State University, Pullman, Washington 99164-7040, USA; 6 USDA/ARS US Meat Animal Research Center, Genetics and Breeding Research Unit, PO Box 166, Clay Center, Nebraska 68933-0166, USA

## Abstract

**Background:**

APOBEC3 (A3) proteins deaminate DNA cytosines and block the replication of retroviruses and retrotransposons. Each *A3 *gene encodes a protein with one or two conserved zinc-coordinating motifs (Z1, Z2 or Z3). The presence of one *A3 *gene in mice (Z2–Z3) and seven in humans, *A3A-H *(Z1a, Z2a-Z1b, Z2b, Z2c-Z2d, Z2e-Z2f, Z2g-Z1c, Z3), suggests extraordinary evolutionary flexibility. To gain insights into the mechanism and timing of *A3 *gene expansion and into the functional modularity of these genes, we analyzed the genomic sequences, expressed cDNAs and activities of the full *A3 *repertoire of three artiodactyl lineages: sheep, cattle and pigs.

**Results:**

Sheep and cattle have three *A3 *genes, *A3Z1*, *A3Z2 *and A3Z3, whereas pigs only have two, *A3Z2 *and A3Z3. A comparison between domestic and wild pigs indicated that *A3Z1 *was deleted in the pig lineage. In all three species, read-through transcription and alternative splicing also produced a catalytically active double domain A3Z2-Z3 protein that had a distinct cytoplasmic localization. Thus, the three *A3 *genes of sheep and cattle encode four conserved and active proteins. These data, together with phylogenetic analyses, indicated that a similar, functionally modular *A3 *repertoire existed in the common ancestor of artiodactyls and primates (*i.e*., the ancestor of placental mammals). This mammalian ancestor therefore possessed the minimal *A3 *gene set, Z1-Z2-Z3, required to evolve through a remarkable series of eight recombination events into the present day eleven Z domain human repertoire.

**Conclusion:**

The dynamic recombination-filled history of the mammalian *A3 *genes is consistent with the modular nature of the locus and a model in which most of these events (especially the expansions) were selected by ancient pathogenic retrovirus infections.

## Background

Mammalian APOBEC3 (A3) proteins have the capacity to potently inhibit the replication of a diverse set of reverse-transcribing mobile genetic elements [1-5]. Susceptible exogenous retroelements include lentiviruses (HIV-1, HIV-2, several strains of SIV and FIV), alpharetroviruses (RSV), betaretroviruses (MPMV), gammaretroviruses (MLV), deltaretroviruses (HTLV), foamy viruses and the hepadnavirus HBV (*e.g*., [6-14]). Susceptible endogenous retroelements include the yeast retrotransposons Ty1 and Ty2, the murine endogenous retroviruses MusD and Pmv, the murine intracisternal A particle (IAP), the porcine endogenous retrovirus PERV and, potentially, extinct elements such as chimpanzee PtERV1 and human HERV-K, all of which require long-terminal repeats (LTRs) for replication [15-23]. In addition, some A3 proteins can also inhibit L1 and its obligate parasite Alu, retrotransposons that replicate by integration-primed reverse transcription [24-30]. An overall theme is emerging in which most – if not all – retroelements can be inhibited by at least one A3 protein.

However, it is now equally clear that the retroelements of any given species have evolved mechanisms to evade restriction by their host's A3 protein(s). For instance, HIV and SIV use Vif to trigger a ubiquitin-dependent degradation mechanism, foamy viruses use a protein called Bet for an imprecisely defined inhibitory mechanism and some viruses such as MPMV, HTLV and MLV appear to employ a simple avoidance mechanism (*e.g*., [6,31-34]). Thus, it appears that all 'successful' retroelements have evolved strategies to resist restriction by the A3 proteins of their hosts.

**Figure 1:**
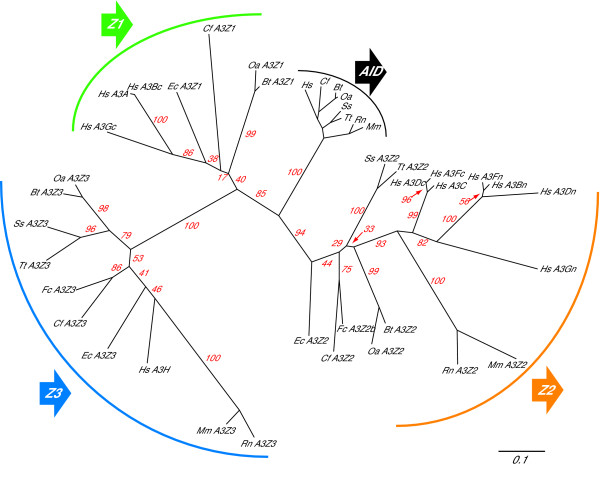
**The mammalian A3 Z domains form three distinct phylogenetic groups**. Bootstrap values are indicated in red. The scale bar represents 0.1 nucleotide changes per codon. See the Methods for details. Abbreviations for mammals: Hs = human, Bt = cow, Oa = sheep, Ss = pig, Tt = peccary, Ec = horse, Cf = dog, Fc = cat, Mm = mouse and Rn = rat. Other abbreviations: n = amino terminal domain and c = carboxy terminal domain.

The defining feature of the A3 family of proteins is a conserved zinc(Z)-coordinating DNA cytosine deaminase motif, H-x_1_-E-x_25–31_-C-x_2–4_-C (x indicates a non-conserved position [35,36]). The A3 Z domains can be grouped into one of three distinct phylogenetic clusters – Z1, Z2 or Z3. (Figure 1 & Additional File 1). The Z-based classification system, proposed originally by Conticello and coworkers [35], was revised recently through a collaborative effort [37]. From hereon, the new A3 nomenclature system will be used. Z1 and Z2 proteins have a SW-S/T-C-x_2–4_-C motif, whereas Z3 proteins have a TW-S-C-x_2_-C motif. Z1 and Z2 proteins can be further distinguished by H-x_1_-E-x_5_-X-V/I and H-x_1_-E-x_5_-W-F motifs, respectively. Z1 proteins also have a unique isoleucine within a conserved RIY motif located C-terminal to the zinc-coordinating residues. At least one protein of each of the Z classes and nearly all identified A3 proteins have exhibited single-strand DNA cytosine deaminase activity. For instance, human A3F, A3G and A3H possess catalytically competent Z2, Z1 and Z3 domains, respectively (*e.g*., [38-41]).

We previously reported a double-domain *A3Z2-Z3 *gene (formerly called *A3F*) from the artiodactyls, sheep (*Ovis aries*), cattle (*Bos taurus*) and pigs (*Sus scrofa*) [42]. However, the fact that mammals have varying numbers of *A3 *genes (*e.g*., 7 in humans and only 1 in mice) led us to wonder whether additional *A3 *genes would be present in artiodactyls. To address this point and to learn more about the evolution and functionality of *A3 *genes in mammals, we sequenced and characterized the full *A3 *repertoire of sheep and pigs. Here, we demonstrated that sheep and cattle actually have three *A3 *genes, *A3Z1*, *A3Z2 *and *A3Z3*, with a conserved potential to encode at least four active and distinct proteins (A3Z1, A3Z2, A3Z3 and A3Z2-Z3). We further showed that porcine lineage has a deletion of the orthologous *A3Z1 *gene and the capacity to encode only three proteins. These data enabled us to deduce that the common ancestor of artiodactyls and primates possessed an *A3 *repertoire consisting of three Z domains (Z1, Z2 and Z3). Our data further suggested an evolutionary model in which most of the human *A3 *gene expansion occurred more than 25 million years ago, during early primate evolution and possibly even associated with pathogen-induced population bottlenecks.

## Results

### Sheep and cattle have three *A3 *genes with a Z1-Z2-Z3 organization

We previously used degenerate PCR, RACE and database mining to identify a cDNA for sheep *A3Z2-Z3 *(formerly called A3F; [42]). However, because humans have seven *A3 *genes and mice have only one, we postulated that artiodactyls such as sheep and cattle might have an intermediate number. To address this possibility unambiguously, we sequenced the entire sheep *A3 *genomic locus. First, a sheep *A3Z2-Z3 *cDNA was hybridized to a sheep BAC library to identify corresponding genomic sequence. Second, hybridization-positive BACS were screened by PCR for those that also contain the conserved flanking genes *CBX6 *and *CBX7*. One BAC was identified that spanned the entire *CBX6 *to *CBX7 *region, and it was sheared, subcloned, shotgun sequenced, assembled and analyzed (Methods).

**Figure 2:**
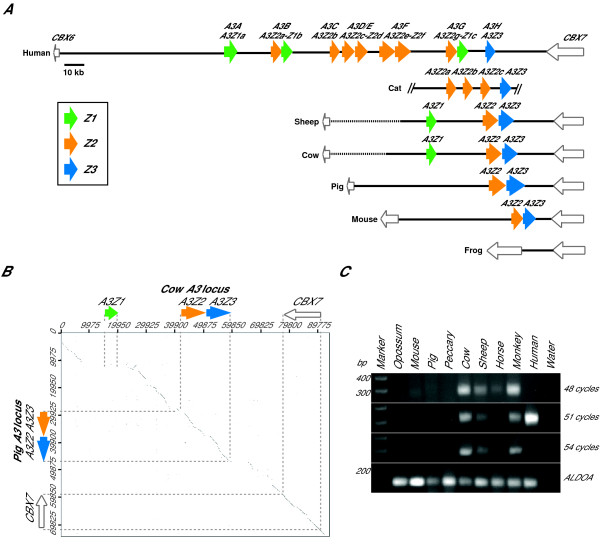
**The *A3 *genomic repertoire of sheep, cattle and pigs**. (*A*) An illustration of the *A3 *genes of the indicated mammals. Z1, Z2 and Z3 domains are colored green, orange and blue, respectively. The conserved flanking genes *CBX6 *and *CBX7 *are shown and the scale is indicated. Solid lines represent finished sequence and dotted lines represent gaps or incomplete regions. Non-mammalian vertebrates such as frogs lack *A3 *genes. (*B*) A dotplot analysis shows A3Z1 in cattle but not in pig genomic sequence. The x- and y-axis numbers designate nucleotide positions within the indicated genomic consensus sequences. (*C*) PCR analysis of genomic DNA from the indicated species showing that a 250–256 bp Z1-specific amplicon can only be obtained from a subset of mammals. The human 51 and 54 cycle amplicons were too abundant to be run on the same gel. Monkey genomic DNA is from the African green monkey. The *ALDOA *gene was used as a positive control (115 bp).

DNA sequence analyses revealed that the sheep genomic locus contained another *A3 *gene between *CBX6 *and *A3Z2 *(Figure 2A). This gene was called *A3Z1*, because it had sequence characteristics of a Z1-type A3 protein. We therefore concluded that sheep have three *A3 *genes and, importantly, that each mammalian *A3 *Z-type was present. This conclusion was supported by the bovine genome assembly, which was released during the course of our studies and showed that cattle also have a sheep-like, three gene *A3 *repertoire (Figure 2A; Btau_4.0 ). The predicted *A3Z1 *coding sequences of sheep and cattle are 86% identical, consistent with the fact that these two ruminant artiodactyls shared a common ancestor approximately 14–25 million years ago (MYA) [43-45].

### The pig has two *A3 *genes with a Z2–Z3 organization

PCR reactions failed to identify an *A3Z1*-like gene in pigs. Since pigs and cattle/sheep last shared a common ancestor approximately 70–80 MYA [43,44], we considered the possibility that the negative PCR result was not a technical failure and that pigs might actually have a different *A3 *repertoire. Again, to unambiguously address this possibility, the pig *A3 *genomic locus was sequenced in entirety. A porcine BAC library was probed with pig *A3Z2-Z3 *cDNA and two hybridization-positive BACS were shotgun sequenced. The sequence assemblies revealed that pigs have only two *A3 *genes *A3Z2 *and *A3Z3 *between *CBX6 *and *CBX7 *(Figure 2A).

The cattle, sheep and pig *A3 *locus genomic sequences were compared using dotplot analyses (Figure 2B & Additional File 2). A 22 kb discontinuity was detected between the cow and the pig sequences. The sheep and pig genomic sequences aligned similarly. Multiple (likely inactive) retroelements were found to flank *A3Z1 *in sheep and cattle. Two were particularly close to the ends of the 22 kb *A3Z1 *region, a LINE/L1 and a SINE/tRNA-Glu. It is possible that one of these elements mediated a simple direct repeat recombination event that deleted the *A3Z1 *region in pigs. However, we were unable to identify such a causative retroelement in the pig genomic sequence.

To begin to address whether the potential *A3Z1 *deletion in pigs occurred recently (*e.g*., a rare deletion fixed by selective breeding) or whether it was more ancient, we asked whether a non-domesticated, distant relative of the pig, the collared peccary (*Tayassu tajacu*), has an *A3Z1 *gene. Lineages leading to present-day domesticated pigs and the peccary diverged approximately 25–35 MYA [43]. A pan-species, *A3Z1 *PCR primer set was developed and used in these experiments. In contrast to human, African green monkey, horse, cow and sheep genomic DNA which yielded a 250–256 bp Z1-specific PCR products confirmable by DNA sequencing, the genomic DNA of domesticated pig, the collared peccary, mice and opossum failed to yield a product even after 54 cycles (Figure 2C). A highly conserved gene, *ALDOA*, was used as a PCR control to demonstrate the integrity of the genomic DNA samples.

Interestingly, Z1 PCR product sequencing and recently released EST sequences revealed that the related hoofed mammal, the horse, also has a Z1-type *A3 *gene (Figure 1C & Additional File 3). Two-'toed' hoofed animals such as sheep, cattle and pigs belong to the ungulate order artiodactyla (even-toe number), and one-'toed' hoofed animals such as horses belong to the ungulate order perissodactyla (odd-toe number). Since these two ungulate orders diverged approximately 80–90 MYA [43,44] and both have species with Z1-type *A3 *genes, it is highly likely that the common ancestor also had an *A3Z1 *gene (as well as *A3Z2 *and *A3Z3 *genes). It is therefore highly unlikely that an *A3Z1 *gene independently appeared at the same genomic position in artiodactyls, perissodactyls and primates. Rather, all of the data support a model where a common ancestor of the domesticated pig and the collared peccary experienced a 22 kb deletion that resulted in the loss of *A3Z1 *(*i.e*., a divergent evolutionary model). Furthermore, since artiodactyls, perissodactyls and humans shared a common ancestor approximately 80–120 MYA [43,44], the presence of Z1-type *A3 *genes in both the primate and the artiodactyl limbs of the mammalian tree is also most easily explained by common ancestry. Thus, our combined datasets indicated that this ancestor possessed a full *A3 *Z repertoire, with one of each type of Z domain (Z1, Z2 and Z3), the minimal substrate required to evolve into the present-day eleven Z domain human *A3 *locus (discussed further below).

### The artiodactyl *A3Z2 *and *A3Z3 *genes combine to encode 3 distinct mRNAs and proteins

**Figure 3:**
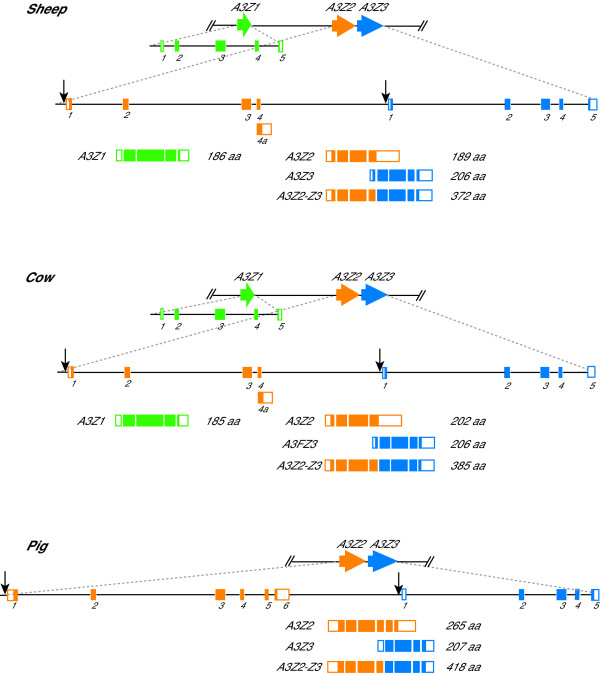
**The coding potential of the sheep, cow and pig *A3 *genes**. Z1, Z2 and Z3 domains are colored green, orange and blue, respectively. The exons are shown below the gene schematics with coding regions represented by filled boxes and untranslated regions by open boxes. The gene schematics and exon blow-ups are drawn to scale. Arrows indicate approximate positions of predicted ISREs (Additional File 4). See the main text and the Methods for details.

We previously characterized several activities of the double-domain A3Z2-Z3 protein from cattle, sheep and pigs [42]. While re-confirming the 5' and 3' ends of the *A3Z2-Z3 *transcripts by RACE, we discovered two interesting variants that were conserved between these three species. First, using sheep and cattle PBMC or cell line cDNA (FLK and MDBK, respectively), 3' RACE frequently produced a smaller than expected fragment. The sequence of this fragment indicated the existence of a short 1037 bp transcript due to premature termination 329 or 330 nucleotides into intron 4 for sheep and cattle, respectively (Figure 3). This truncated transcript was readily amplified from sheep and cattle PBMCs and represented by existing EST sequences (Additional File 3 and data not shown). Therefore, this novel transcript was predicted to result in a single-domain Z2 protein, A3Z2, with a length of 189 and 202 amino acids for sheep and cattle, respectively (Figure 3 & Additional File 3). A pig A3Z2 transcript was also identified by RACE and EST sequences but, in contrast to sheep and cattle, exon 4 was spliced to two additional exons before terminating prematurely (Figure 3 & Additional File 3). As a consequence, pig A3Z2 was predicted to be 265 amino acids. These analyses indicated that artiodactyls have the capacity to express a single domain A3Z2 protein, in contrast to what we had deduced previously [42].

Second, 5' RACE data and cattle and pig EST sequences suggested that yet another mechanism served to broaden the coding potential of the artiodactyl *A3 *locus (Additional File 3 and data not shown). Several transcripts appeared to originate from the region immediately upstream of *A3Z3*, whereas our prior studies had only detected transcripts originating upsteam of *A3Z2 *[42]. A comparison of cDNA and genomic sequences revealed the presence of an exon in this location (*A3Z3 *exon 1 in Figure 3). Transcripts initiating here produced 941 (sheep), 964 (cow) or 1003 (pig) nucleotide messages. The resulting A3Z3 protein was predicted to be 206 residues for sheep and cattle and 207 for pigs (Figure 3).

The *A3Z3 *mRNA data strongly suggested the existence of an internal promoter. This was supported by cis-regulatory element prediction algorithms, which identified a conserved interferon-stimulated response element (ISRE) upstream of *A3Z3*, as well as upstream of *A3Z2 *(Figure 3 & Additional File 4). These ISREs were strikingly similar to those located in the promoter regions of human *A3DE*, *A3F *and *A3G*, supporting the likelihood that interferon-inducibility is a conserved feature of many mammalian *A3 *genes (*e.g*., [8,46-49]). These putative ISREs significant similarity to functional elements in known interferon-inducible genes *ISG54 *and *ISG15 *[50-52]. We also predicted binding sites for another well-known transcription factor, Sp1, upstream of the *A3Z3 *transcription start site. This activator was also recently reported for human and cat *A3 *genes ([53,54]; LaRue & Harris, data not shown).

Together with our previous data on the double domain A3 protein of these artiodactyl species, A3Z2-Z3 [42], these expression and promoter data revealed that two single-domain *A3 *genes can readily encode at least three distinct proteins – A3Z2, A3Z3 and A3Z2-Z3. A similar strategy may also be used by rodents, which also have an *A3 *gene with Z2 and Z3 domains. A similar modularity was reported recently for the cat *A3 *locus, where two single domain *A3 *genes combined to produce a functional double-domain A3 protein [54]. We suggest that combining single-domain A3s to yield functionally unique double-domain proteins may be a general strategy used by many mammals to bolster their A3-dependent innate immune defenses.

### All four artiodactyl A3 proteins – A3Z1, A3Z2, A3Z3 and A3Z2-Z3 – elicit DNA cytosine deaminase activity

All currently described A3 proteins have elicited single-strand DNA cytosine to uracil deaminase activity in one or more assays (*e.g*., [24,41,42,54-59]). For instance, we showed that the artiodactyl A3Z2-Z3 proteins could catalyze the deamination of *E. coli *DNA and retroviral cDNA [42]. However, catalytic mutants indicated that only the N-terminal Z2 domain of cow, sheep and pig A3Z2-Z3 was active. This observation contrasted with data for the double-domain human A3B, A3F and A3G proteins, where the C-terminal domain clearly contains the dominant active site (*e.g*., [30,38-40,42,60]). Nevertheless, these datasets suggested that the double-domain A3 proteins have separated function, with one domain predominantly serving as a catalytic center and the other as a regulatory center.

**Figure 4:**
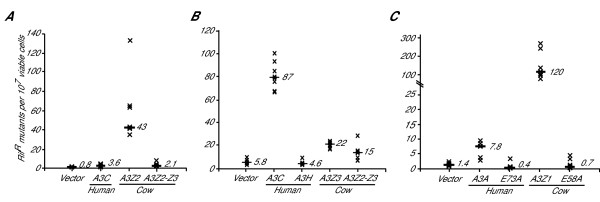
**The artiodactyl A3 proteins catalyze DNA cytosine deamination**. (*A*) Cow A3Z2 triggers a strong mutator phenotype in *E. coli*. Rif^R ^mutation frequency of 4–6 independent bacteria cultures expressing basal levels of indicated A3 proteins (each X represents data from a single culture). To facilitate comparisons, the median mutation frequency is indicated for each condition. (*B*) Cow A3Z3 triggers a modest mutator phenotype in *E. coli*. Labels and conditions are similar to those in panel (A), except IPTG was used to induce protein expression. (*C*) Non-induced cow A3Z1 triggers a strong mutator phenotype in *E. coli*, which is completely abrogated by substituting the catalytic glutamate (E58) for alanine. Labels are similar to those in panel (A).

However, a recent study with human A3B indicated that both Z domains have the potential to be catalytically active [61]. It was therefore reasonable to ask whether the single domain A3Z2 and A3Z3 proteins of artiodactyls would be capable of DNA cytosine deamination in an *E. coli*-based activity assay. Elevated frequencies of rifampicin-resistance (Rif^R^) mutations in *E. coli *provide a quantitative measure of the intrinsic A3 protein DNA cytosine deaminase activity (*e.g*., [38,40,56,57]). In contrast to full-length cow A3Z2-Z3, which triggered a modest 2-fold increase in the median Rif^R ^mutation frequency over the vector control, non-induced levels of cow A3Z2 caused a large 50-fold increase (Figure 4A). The pTrc99-based vector used in these studies has an IPTG-inducible promoter, and induced levels of cow A3Z2 prevented *E. coli *growth, presumably through catastrophic levels of DNA cytosine deamination. In contrast, induced levels of sheep or pig A3Z2 proteins were not lethal, but their expression also caused significant increases in the median Rif^R ^mutation frequency (Additional File 5 and LaRue & Harris, data not shown). Thus, as anticipated by our prior studies, the A3Z2 proteins of cattle, sheep and pigs showed intrinsic DNA cytosine deaminase activity.

We were therefore surprised that induced levels of the cow single-domain protein A3Z3 also caused a significant 4-fold increase in the median Rif^R ^mutation frequency (Figure 4B). This result contrasted with the related Z3 protein of humans, A3H, which appeared inactive in this assay (Figure 4B & Additional File 3). However, it is worth noting that other Z3-type A3 proteins, a different human A3H variant, African green monkey A3H, rhesus macaque A3H and cat A3Z3 (formally A3H), all showed evidence for DNA deaminase activity in the *E. coli*-based mutation assay and/or in retrovirus infectivity assays [41,54,62,63]. Thus, our intended human A3H control appears to be the exception rather than the rule and that the single-domain A3Z3 protein of artiodactyls is capable of DNA cytosine deaminase activity.

We also observed that the artiodactyl A3Z1 protein was capable of robust DNA cytosine deaminase activity (*e.g*., Figure 4C and LaRue and Harris, unpublished data). This result was fully anticipated based on the fact that the related Z1 domain proteins of humans A3A, A3B and A3G are catalytically active [24,30,61,64]. However, it is worth noting three observations suggesting that cow A3Z1 is the most active of all reported A3 proteins. First, we were never able to directionally clone (even non-induced) *A3Z1 *of sheep or cattle into pTrc99A, which has a leaky promoter. Second, we were only able to topoisomerase-clone cow *A3Z1 *in a direction opposite to the *lac *promoter (n > 12). Finally, even with cow A3Z1 in the promoter-opposing orientation in the topoisomerase cloning plasmid, we observed 100-fold increases in Rif^R ^mutation frequency in the *E. coli*-based mutation assay that were fully dependent on the catalytic glutamate E58A (presumably due to expression from a cryptic promoter; Figure 4C). To summarize this section, all four of the A3 proteins of artiodactyls demonstrated intrinsic DNA cytosine deaminase activity.

### A3Z1, A3Z2, A3Z3 and A3Z2-Z3 differentially localize in cells

**Figure 5:**
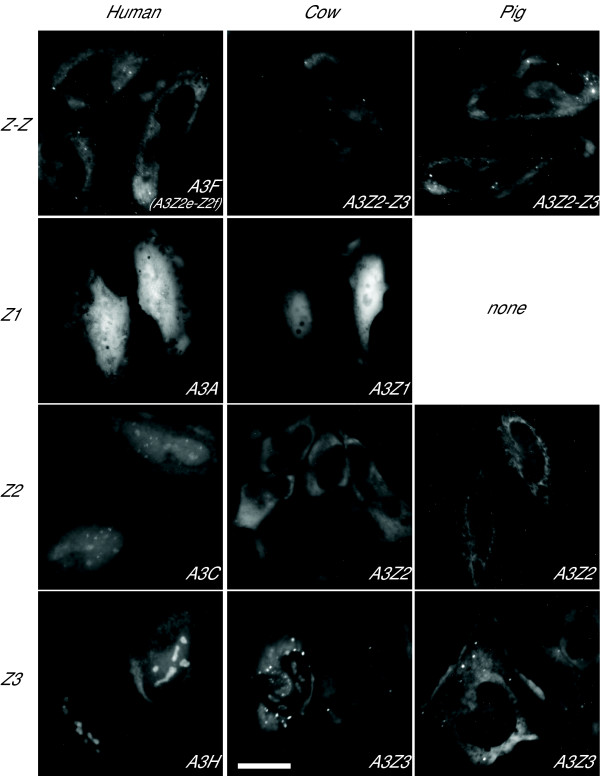
**The subcellular distribution of cow and pig A3 proteins in comparison to human A3 proteins with similar Z domains**. Representative images of live HeLa cells expressing the indicated A3-GFP fusion proteins are shown. The scale bar represents 25 μm.

Fluorescent microscopy was used to examine the subcellular distribution of each of the artiodactyl A3 proteins fused to GFP. Like the human A3 proteins, which each have unique overall subcellular distributions, we imagined that distinct localization patterns might correlate with differential functions. For instance, the first column of Figure 5 shows representative images of live HeLa cells expressing human A3F-GFP, A3A-GFP, A3C-GFP and A3H-GFP, which predominantly localize to the cytoplasm, cell-wide with a nuclear bias, cell-wide and cell-wide with a clear nucleolar preference, respectively. Cow A3Z1-GFP showed an indiscriminate cell-wide distribution similar to that of human A3A-GFP and GFP alone (Figure 5, second row and data not shown).

As shown previously, cattle and pig A3Z2-Z3-GFP localize to the cytoplasm, with some cells showing bright aggregates (Figure 5, row 1; [19,42]). Cattle and pig A3Z2 also appeared predominantly cytoplasmic, but a significant fraction clearly penetrated the nuclear compartment (row 3). The subcellular distribution of cattle and pig A3Z2 differed from the similarly sized Z2 protein human A3C, which was cell-wide, and it is therefore likely that an active process underlies the cytoplasmic bias of the artiodactyl A3Z2 proteins. Interestingly, the A3Z3 proteins of cattle and sheep, like human A3H, localized cell-wide with clear accumulations in the nucleoli (row 4). Similar data were obtained using these GFP fusion constructs in live cattle MDBK cells and in live pig PK15 cells (LaRue & Harris, data not shown). These fluorescent microscopy observations demonstrated that all of the artiodactyl A3 proteins can be expressed in mammalian cells and that they have both distinct and overlapping subcellular distributions.

### The artiodactyl *A3 *genes show evidence for positive selection

Many human, non-human primate and feline *A3 *genes show signs of strong positive selection, which can be interpreted as evidence for a history filled with pathogen conflicts [41,54,65,66]. However, given the relative stability of the artiodactyl *A3 *locus, at least in terms of gene number, we wondered whether the artiodactyl *A3 *genes might be under less intense selective pressure (perhaps even neutral or negative). This possibility was assessed using two methods to compare the number of mutations that resulted in amino acid replacements to the number that were silent between pairs of artiodactyl species. This ratio of replacement (dN) to silent (dS) mutations yields an omega (ω) value, which if greater than one is indicative of positive selection, if equal to one of neutral selection and if less than one of negative selection. We focused these analyses on the single exon that encodes the conserved Z domain to minimize potentially confounding effects from recombination.

We first generated a combined phylogeny for each distinct *A3 *Z domain and its inferred ancestral sequences (Additional File 6). Using the PAML free ration model, the artiodactyl *A3Z1 *and the *A3Z2 *genes appeared to be under a weak negative selection pressure, with ω values uniformly below one (Additional File 6). Similarly, since the existence of the last common ancestor of cattle and sheep or of the pig and peccary, the artiodactyl *A3Z3 *genes showed evidence for weak negative selection pressure (Additional File 6C). However, a comparison of the inferred ancestral ruminant sequence with the inferred porcine sequence yielded a ω value of 1.5, suggesting that the ancestor(s) of modern day artiodactyls may have experienced intermittent positive selection (Additional File 6C). These values were not as high as those for primate *A3Z3 *(*A3H *data originally reported by [41] and re-calculated here with a representative clade shown in Additional File 6C). Moreover, all of these data contrasted sharply with the artiodactyl and primate *AID *genes, which are under an obvious strong negative selection pressure presumably for essential functions in antibody diversification.

However, because the free ratio model averages all possible sites and has a tendency to underestimate instances of positive selection, we subsequently used PAML NsSites to do a more focussed examination of artiodactyl *A3 *Z domain variation. Several distinct selection models were used (M2 and M8 and two codon frequency models F61 and F3 × 4), and each yielded significant signs of positive selection (Table 1; see Methods for procedural details and Additional File 3 for sequence information). The Z3 domain *A3 *genes of sheep, cattle, pig, peccary and horse showed the highest dN/dS ratios, ranging from 4.4 to 5.8 and indicating that 22–31% of the residues were subjected to positive selection. Lower but still significant positive dN/dS ratios were obtained for the Z2 domain *A3 *genes (1.7 to 2.3 with 33 to 46% of the residues under positive selection). Moreover, together with available dog and horse Z1 sequences, the Z1 *A3 *genes of cattle and sheep showed intermediate degrees of positive selection, with dN/dS ratios of 2.5 to 3.9 and 28 to 50% of the residues under some degree of positive selection (Table 1 & Additional File 3). Thus, similar to most other mammals analyzed to date, the artiodactyl *A3 *genes have been subjected to strong evolutionary pressure (see Discussion).

**Table 1:** Evidence for positive selection in the artiodactyl Z domains.

**Z domain**^a^	**Codon frequency model**^b^	**Comparison of null and positive selection models**^c^	**Significance**	**Tree length**^d^	**dN/dS (%)**^e^
Z1	F61	M1–M2	p = 0.01	2.6	2.5 (50)
		M7–M8	p = 0.01	2.6	2.5 (50)
	F3 × 4	M1–M2	p = 0.04	3.9	3.9 (28)
		M7–M8	p = 0.02	3.9	3.9 (33)
Z2	F61	M1-M2	p = 0.005	2.4	2.3 (46)
		M7–M8	p = 0.004	2.4	2.3 (45)
	F3 × 4	M1–M2	p = 0.3	3.0	1.7 (27)
		M7–M8	p = 0.04	3.0	1.7 (33)
Z3	F61	M1–M2	p < 0.001	2.4	4.5 (30)
		M7–M8	p < 0.001	2.4	4.4 (31)
	F3 × 4	M1–M2	p < 0.001	3.1	5.8 (22)
		M7–M8	p < 0.001	3.1	5.7 (23)

^a^Only the sequences of Z domain-encoding exons were used in these analyses (see **Table S1 **for GenBank accessions).

^b^Two different codon frequency models were used to minimize potentially artificial results.

^c^Likelihood ratio tests were done to compare the null models M1 and M7 to each positive selection model M2 and M8, respectively, using PAML NsSites.

^d^Tree length provides a measure of nucleotide substitutions per codon along all combined phylogenetic branches.

^e^The percentage of all codons influenced by positive selection is indicated in parentheses.

### *A3 *Z domain distribution in mammals

**Figure 6:**
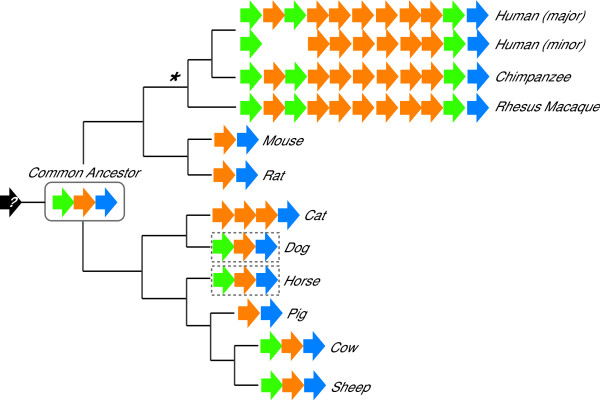
**The distribution of *A3 *Z domains in mammals**. The common ancestor of the indicated placental mammals was inferred to have a Z1-Z2-Z3 *A3 *gene repertoire. Z1, Z2 and Z3 domains are colored green, orange and blue, respectively. A question mark specifies the original *AID*-like ancestor. An asterisk indicates the likely period in which the bulk of the primate *A3 *gene expansion occurred (see main text, Figure 7 and Additional File 7). Some humans are A3B deficient (minor allele; [75]). The boxed *A3 *Z domain repertoires constitute the minimal set inferred from incomplete genomic sequences and EST data (Additional File 3).

Our studies strongly indicated that the present-day *A3 *locus of sheep and cattle resembles one that existed in the common ancestor of placental mammals, consisting of precisely one of each of the three phylogenetically distinct Z domains: Z1, Z2 and Z3 (Figure 6; also see Figure 1 & Additional File 3). Molecular phylogenetic data helped us infer that such a common ancestor existed approximately 100–115 MYA [43,44]. However, the bulk of the primate *A3 *gene expansion most likely occurred more recently because the main branches leading to rodents and humans split 90–110 MYA. It is therefore likely that rodents lost a Z1 *A3 *gene after branching off of the main mammalian tree (like pigs, cats and some humans; see Figure 6 &**Discussion**). Moreover, the recently published draft of the rhesus macaque genome helped to further whittle-down when the bulk of the primate-specific expansion occurred, because these animals also possess a human-like *A3 *gene repertoire (Figure 6; [41,67,68] and our unpublished data). Thus, since the human and macaque lineages diverged approximately 25 MYA [43,67,69], the massive expansion from the inferred sheep/cow-like Z1-Z2-Z3 *A3 *gene set to a locus resembling the present-day human repertoire must have occurred within a relatively short 65–85 million year period (indicated by an asterisk in Figure 6).

### A minimum of 8 recombination events were required to generate the present-day human *A3 *locus from the common ancestor of artiodactyls and primates

The inferred ancestral Z1-Z2-Z3 locus was used as a starting point to deduce the most likely evolutionary scenario that transformed it into the much larger eleven Z domain human *A3 *repertoire. Two types of recombination events were considered, tandem duplications (obviously required for *A3 *gene expansion) and deletions. Self-similarities in the DNA sequence of the human *A3 *locus provided strong evidence for prior tandem duplications by unequal crossing-over (for more details on tandem duplication modeling see [70,71]). This mode of evolution is also supported by the fact that the human *A3 *locus contains many retroelements that could serve as substrates for homologous recombination [35]. Since our present studies showed that the Z domains are highly modular and capable of individual function, they were considered as the core units for duplications in our inference procedures (*i.e*., an unequal cross-over event can simultaneously duplicate one or more tandemly arranged Z-domains and associated flanking sequences). Similarly, deletions could involve one or more Z domains and result from unequal crossing-over or intra-chromosomal events.

**Figure 7:**
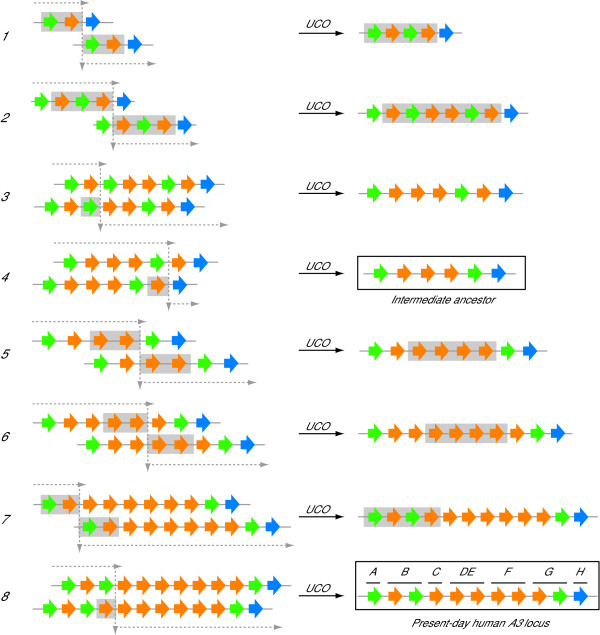
**An 8-event model for the duplication and deletion history of the human *A3 *repertoire**. Z1, Z2 and Z3 domains are colored green, orange and blue, respectively. Five duplication and three deletion events were predicted to transform the ancestral locus into the present-day human *A3 *repertoire. The first event was predicted to occur between two copies of the ancestral Z1-Z2-Z3 locus. The Z domain(s) affected by each unequal crossing-over (UCO) event is shaded gray. The crossing-over points are indicated by a dashed line arrows, and the resulting Z domain configurations are shown (we assumed that new configurations achieved homozygosity prior to being involved in a subsequent UCO). Although deletion events 3 and 4 are illustrated as interchromosomal UCOs, they could have also been caused by intrachromosomal events. Event 4 is depicted before an inferred 'intermediate ancestor' common to nearly all of our models and therefore considered parsimonious, but this event could have occurred any time after event 2. The underlying phylogeny for this model is identical to that shown in Figure 1, except the N-terminal domain of human A3B diverged prior to the point at which the N-terminal domains of human A3F/A3DE and A3G split. An alternative depiction of this model is shown in Additional File 7 and details can be found in the main text and Methods.

An 8-event model for human *A3 *Z domain history is shown in Figure 7 (see Additional File 7 for an alternative representation). This model can be appreciated by considering the present-day human locus and then working backward in time using highly similar local sequences within the *A3 *locus, which provide 'footprints' for recent recombination events. First, full-length *A3A *and the Z1 domain of *A3B *are 97% identical, and they are flanked by nearly homologous ~5.5 kb regions (*i.e*., direct repeats of 95% identity). These footprints strongly suggested that a recent duplication of two consecutive ancestral domains (Z1–Z2) gave rise to present-day *A3B *(event 7). Second, we inferred that this recent duplication resulted in a vestigial Z2 domain upstream of *A3C*, which was subsequently deleted prior to the divergence of human and chimpanzee lineages (event 8). Such a deletion event was supported by the fact that ~3 kb regions of 92% identical DNA reside upstream of the present-day *A3B *and *A3C *Z2 domains (these repeats lack similarity to other DNA within the locus). Third, a 92% similarity between two regions (~10 kb) encompassing the *A3DE *and *A3F *genes suggested they originated from a recent duplication. Moreover, a similar level of identity was found between two other regions (~10 kb) encompassing the Z2 domains of *A3F *and *A3G*. This strongly supported a common ancestral origin for the N-terminal domains of the *A3DE*, *A3F *and *A3G *genes (events 5 and 6). The likelihood of these four relatively recent events suggested that the ancestral locus configuration prior to event 5 [Z1-(Z2)_3_-Z1–Z3] was a key intermediate in the evolution of the primate *A3 *locus (event 4 product in Figure 7).

Unequal crossing-over events prior to the ancestral intermediate were harder to infer because the footprints have been erased by sequence divergence. We therefore developed an algorithm to compute the minimal series of duplication and deletion events that could have generated this intermediate locus from the Z1-Z2-Z3 ancestor. Three minimal scenarios were found and each involved 4 events. However, when phylogenetic data were considered, only one scenario was plausible and it involved a 2-domain duplication, a 3-domain duplication and two single domain deletions (respectively, events 1 to 4 in Figure 7 & Additional File 7). Thus, together with the events detailed above, we inferred that the current human *A3 *repertoire is the product of 8 recombination events – 5 duplications and 3 deletions.

Theoretically, models with as few as 5 events are possible if the likely intermediate locus configuration is ignored. However, these models are also untenable as they clash with phylogenetic and local sequence alignment data. It should be noted that 8 events represent only a lower bound to explain the evolution of the *A3 *human locus. Scenarios involving more than 8 events could also lead to the same domain organization, and some events may have left no observable trace in the human lineage. Thus, this lower bound could increase when the complete *A3 *locus sequence of more mammals, and especially more primates, comes available. Finally, it is worth emphasizing that most (if not all) of the 8 recombination events modeled here happened in the 65 to 85 million year period between the points when the rodent and Old World monkey (*e.g*., rhesus macaque) lineages split from the phylogenetic branch that led to humans (the time frame indicated by the asterisk in Figure 6).

## Discussion

The present studies were initiated to gain a better understanding of the full *A3 *repertoire of three artiodactyl lineages – cattle, pigs and sheep – and to achieve insights into the mechanism and timing of the *A3 *gene expansion in mammals. We demonstrated that sheep and cattle have three *A3 *genes, *A3Z1 A3Z2 *and *A3Z3. *However, the latter two genes and their counterpart in pigs have the unique ability to produce a double-domain protein A3Z2-Z3, in addition to single-domain polypeptides. Thus, the A3 proteome of these species is more formidable than gene number alone would indicate. Our studies also help highlight the important point that, although A3 proteins consist of either one or two conserved Z domains, each of these domains can function and evolve independently.

Prior to the present studies, it was clear that most (if not all) placental mammals had Z2- and Z3-type *A3 *domains (*e.g*., human, mouse, cat, pig, sheep and cow [35,36,42,54,72]). It was far less clear how broadly the Z1 domain distributed. Here, we presented two critical lines of evidence strongly indicating that the Z1 distribution is equally broad and, importantly, that the common ancestor of placental mammals had a Z1-Z2-Z3 *A3 *gene repertoire, similar to that of present-day sheep and cattle. First, the sheep and cattle *A3 *genomic sequences demonstrated the presence of a Z1-type *A3 *gene outside of the primate phylogenetic branches (Figure 6). Second, our pan-species Z1 PCR data, public EST data and draft genomic sequences from horses and dogs combined to show that a *A3Z1 *gene exists in other parts of the artiodactyl-containing phylogentic branch set. These data supported a model in which the common ancestor of the primate- and the artiodactyl-containing mammalian super-orders, Euarchontoglires and Laurasiatheria, respectively, had a *A3Z1 *gene and precisely one of each of the three conserved Z domain types (*i.e*., a divergent model for *A3 *gene evolution, as opposed to one in which *A3Z1 *genes evolved independently in several limbs of the mammalian tree). We have therefore established a critical foundation for understanding the function(s) and evolutionary history of the *A3 *repertoire of any other placental mammal.

It is noteworthy that our pan-species Z1 PCR analyses failed to generate product from opossum genomic DNA and that the recently released opossum and platypus genomic sequences lack *A3 *genes (Figure 2C; [73,74]). This is unlikely to be a gap in the DNA sequence assemblies because, like non-mammalian vertebrates, DNA and protein searches clearly revealed the *A3*-flanking genes *CBX6 *and *CBX7 *in both animals (LaRue & Harris, unpublished data). Thus, unfortunately, these two interesting non-placental mammals are unlikely to provide significant insights into the earliest stages of *A3 *gene evolution (*i.e*., pre-dating the Z1-Z2-Z3 ancestor described here). Perhaps data from the other two placental mammal super-orders, Afrotheria and Xenarthra (*e.g*., represented by animals such as aardvarks and anteaters, respectively), will help shed light on earlier stages of *A3 *gene evolution, when presumably an *AID*-like gene transposed between *CBX6 *and *CBX7 *and duplicated to give rise to the ancestral Z1-Z2-Z3 locus. Nevertheless, because all current data indicate that the *A3 *genes are specific to placental mammals, we hypothesize that a unique role of these genes may relate to the placenta itself, where the A3 proteins may function to help protect the developing fetus from potentially harmful retrotransposition events and/or retroviral infections.

A growing body of evidence indicates that the sole function of the *A3 *genes of mammals is to provide an innate immune defense to retrovirus and retrotransposon mobilization. This is supported by the fact that the single *A3 *gene of mice is dispensable and that many of the mammalian *A3 *genes show evidence for a strong diversifying selection ([10,41,65,66] and this study, Table 1). Although the reason(s) are presently unknown, a large *A3 *repertoire is clearly more important for some mammals than it is for others. Humans, chimpanzees and rhesus macaques have 11 Z domains, approximately 3- to 4-fold more than any other known non-primate mammal (Figure 6). Indeed, our studies indicated that the ancestors of humans and chimpanzees experienced at least eight Z domain recombination events, which is more than the total combined number of events for other known mammals. Therefore, despite the fact that the artiodactyl *A3 *genes show evidence for positive selection, their relative stability in copy number suggests that a considerable disadvantage – such as the potential to mutate genomic DNA – may outweigh the innate benefit of having numerous *A3*s to combat potentially invasive retroelements. This possibility may very well relate directly to an emerging trend in mammals, which is the frequent loss of a *A3Z1 *gene which encodes a protein that can penetrate the nuclear compartment (*e.g*., Figure 5). An *A3Z1 *deletion was shown here for pigs, inferred here for cats and mice/rats, and demonstrated recently for some human populations (Figure 6 and [75]).

Finally, a major question is what selective pressure(s) drove the *A3 *expansion from an ancestral Z1-Z2-Z3 repertoire to the present day human Z1-Z2-Z1-(Z2)_6_-Z1-Z3 repertoire? We propose that large-scale events such as gene expansions were selected by extremely pathogenic or lethal retroviral epidemics, because rare expansions would have been easily lost amongst a population of non-expanded alleles. A powerful selective pressure such as a lethal epidemic has the potential to produce a population bottleneck such that mostly (or only) pathogen-resistant individuals would survive (*i.e*., those with the appropriate disease-resistant *A3 *repertoire). Such powerful selective pressures would have the potential to promote and perhaps even cause speciation events. We further predict that such events may be marked by changes in *A3 *Z domain copy number. It is therefore quite plausible that at least some of the eight recombination events required to transform the ancestral Z1-Z2-Z3 repertoire into the present day human Z1-Z2-Z1-(Z2)_6_-Z1-Z3 repertoire may have protected our human ancestors from ancient retroviral infections and thereby facilitated the evolution of primates (a process that we have termed primatification).

## Conclusion

The *A3 *locus of sheep and cattle consists of three genes, *A3Z1*, *A3Z2 *and *A3Z3*, and the potential to encode four functional proteins, three directly and one (A3Z2-Z3) by read-through transcription and alternative splicing. The *A3 *locus of pigs experienced a deletion and therefore lacks *A3Z1*. The artiodactyl *A3 *repertoire demonstrates a unique modularity centered upon the conserved zinc-coordinating motifs. DNA deaminase activity data and subcellular localization studies suggest that this modularity may also correspond to a broader functionality. All of the data combined to indicate that the common ancestor of artiodactyls and humans possessed a sheep/cattle-like *A3 *gene set, with the organization and capacity to evolve into the present day repertoires. The remarkable *A3 *gene expansion in the primate lineage – from the three ancestral genes (*A3Z1*-*A3Z2*-*A3Z3*) to the present-day eleven Z-domain human repertoire – was predicted to require a minimum of eight recombination events, most of which may have been required to thwart an ancient retroviral infections.

## Methods

### Genomic DNA sequences

A combination of array hybridization, *A3-*, *CBX6*- and *CBX7*-specific PCR was used to identify one *A3*-positive BACs for sheep (CHORI-243 clone 268D23; a kind gift from P. de Jong, BACPAC Resources Center, ) and two for pigs (RPCI-44 clones 344O17 and 408D3; [76]). *E. coli *were transformed with these BACs, grown to saturation in 50 ml cultures and used for DNA preparations as recommended (Marligen Biosciences). Purified BAC DNA was sheared to an average of approximately 3000 bp (Hydroshear method, Genomic Solutions). Fragment ends were blunted with T4 and Klenow DNA polymerases (NEB) and ligated into pBluescriptSK- (Stratagene) or pSMART-HC (Lucigen). Individual subclones were picked randomly and sequenced (ABI3730; Applied Biosystems). Phrap (P. Green, 1996, ) and Sequencher 4.8 (Gene Codes Corp.) were used to assemble DNA sequences and they were groomed manually. Sequence coverage for the sheep *A3 *locus averaged 4.5 sequences and the pig 27 sequences. The genomic sequences were compared using Jdotter software (; [77]). Repetitive sequences were identified using RepeatMasker .

*A3 *exons were identified by directly comparing the genomic DNA sequences with cDNA, EST and RACE sequences (below, Additional Files 3 &8 and [42]). Predicted ISREs were identified and compared using the TransFac and Biobase databases through the softberry NSITE portal . The sheep *CBX6 *exons were identified with the help of GenBank EST sequences EE808826.1, DY519385.1 and EE822736.1. The pig *CBX6 *exons were also identified in this manner using BP158234.1, BP997823.1 &BP153834.1. The sheep and pig *CBX7 *exons were identified by homology to the cow gene (below). Other *CBX6 *and *CBX7*, sequences, respectively, were NM_014292.3 and NM_175709.2 (human), NM_001103094 and XM_604126 (cow), NM_028763.3 and NM_144811 (mouse) and NM_001016617.2 &NM_001005071 (frog).

### *A3Z1 *gene degenerate PCR analyses

Genomic DNA was isolated from the following tissues or cell lines: opossum kidney tissue, mouse NIH-3T3 cells, pig PK-15 cells, peccary brain tissue, cow MDBK cells, sheep FLK cells, horse blood cells (PBMC), African green monkey COS7 cells and human 293T cells (DNeasy, Qiagen). 10ng genomic DNA was used as template for PCR using primers designed to anneal to all known *A3Z1 *genes: 5'-GCC ATG CRG AGC TSY RCT TCY TGG and 5'-GTC ATD ATK GWR AYT YKG GCC CCA GC-3'. Two PCR rounds were used to achieve the final number of cycles (30 plus 18, 21 or 24 cycles). Amplicons were analyzed by agarose gel electrophoresis, TOPO-cloned (Invitrogen) and subjected to DNA sequencing. In all instances, the expected *A3Z1 *fragments were recovered (*e.g*., Z1 of human *A3A*, *A3B *and *A3G *could all be detected in a single reaction). 30 PCR cycles using identical conditions and degenerate primers for the *ALDOA *gene were used as a positive control (5'-CGC TGT GCC CAG TAY AAG AAG GAY GG-3' and 5'-CTG CTG GCA RAT RCT GGC YTA).

### Identifying expressed mRNAs by RACE

RNA was extracted from fresh pig (*Sus scrofa *Landrance/Yorkshire cross), sheep (*Ovis aries *Hampshire) and cattle (*Bos taurus *Hereford) PBMCs using the QIAamp RNA Blood mini kit (Qiagen). 5' and 3' RACE was performed using reagents from the FirstChoice RLM-RACE kit (Ambion). The protocol was modified slightly by using SAP (Roche) instead of CIP to remove 5'-phosphates. *A3 *cDNA 5' and 3' ends were amplified using Phusion high-fidelity polymerase (NEB), purified and TOPO-cloned (Invitrogen). All *A3*-specific primers used in conjunction with the 5' and 3' RACE primers are listed in Additional File 8.

### A3 expression plasmids

The pTrc99A-based *E. coli *expression plasmids for sheep, cattle and pig A3Z2-Z3 and for human A3C and A3H were reported previously [42,56]. Other pTrc99A-based constructs were made by ligating KpnI- and SalI-digested PCR fragments into a similarly cut vector. Cow A3Z2 and A3Z3 were amplified from PBMC cDNA (above) using primers 5'-NNN NGA GCT CAG GTA CCA CCA TGC AAC CAG CCT ACC GAG GC & 5'-NNN NGT CGA CTC ACC CGA GAA TGT CCT C and 5'-NNN NGA GCT CAG GTA CCA CCA TGA CCG AGG GCT GGG C & 5'-NNN NGT CGA CCT AAA TTG GGG CCG TTA GGA T, respectively. Pig A3Z2 was amplified from the USMARC1 cDNA library [78] using primers 5'-NNN NGA GCT CAG GTA CCA CCA TGG ATC CTC AGC GCC TGA GAC and 5'-NNN NGT CGA CTC AGC GGT AAC AAA TCC.

Cow A3Z1 was a special case (see main text). It was amplified from PBMC cDNA (above) using primers 5'-NNN NGA GCT CAG GTA CCA C CA TGG ACG AAT ATA CCT TCA CT and 5'-NNN NGT CGA CGT TTT GCT GAG TCT TGA G and TOPO-cloned into pCR-BLUNT-II-TOPO (Invitrogen). As a control, human A3A was amplified using 5'-NNN NGA GCT CGG TAC CAC CAT GGA AGC CAG CCC AGC and 5'-NNN NGT CGA CCC CAT CCT TCA GTT TCC CTG ATT CTG GAG and TOPO-cloned. Catalytic mutant derivatives of the cow A3Z1 and human A3A plasmids were constructed by site-directed mutagenesis (Stratagene) using oligonucleotides 5'-CCT GCC ATG CAG CGC TCT ACT TCC TG & 5'-CAG GAA GTA GAG CGC TGC ATG GCA GG and 5'-GGC CGC CAT GCG GCG CTG CGT TCT TG & 5'-CAA GAA GCG CAG CGC CGC ATG GCG GCC, respectively.

The artiodactyl A3 proteins were expressed in Hela cells as N-terminal fusions to eGFP (pEGFP-N3; Clontech). Cow and pig A3Z2-Z3-eGFP and the human A3A-, A3C-, A3F- and A3H-eGFP constructs were reported previously [42,79]. Cow and pig A3Z2-eGFP plasmids were made by cloning SacI/SalI-digested PCR products generated using primers 5'-NNN NGA GCT CAG GTA CCA CCA TGC AAC CAG CCT ACC GAG GC & 5'-NNN NGT CGA CCC CGA GAA TGT CCT CAA G and 5'-NNN NGA GCT CAG GTA CCA CCA TGG ATC CTC AGC GCC TGA GAC & 5'-NNN NGT CGA CCC ACC TGG CGT GAG CAC C, respectively. Cow and pig A3Z3-eGFP plasmids were made similarly using primers 5'-NNN NGA GCT CAG GTA CCA CCA TGA CCG AGG GCT GGG C & 5'-NNN GTC GAC TCC AAT TGG GGC CGT TAG GAT and 5'-NNN NGA GCT CAG GTA CCA CCA TGA CCG AGG GCT GGG CT & 5'-NNN GTC GAC TCC TCT CGA GTC ACT TCT TGA, respectively

Due to the toxicity of cow *A3Z1 *in *E. coli*, an *A3Z1*::intron-eGFP plasmid was made by overlapping PCR to join 3 separate fragments: *A3Z1 *exons 1 and 2 (primers 5'-NNN NGA GCT CAG GTA CCA C CA TGG ACG AAT ATA CCT TCA CT and 5'-CCT GGA CTC ACC TTG TTG CGC), an L1-derived intron ([80]; primers 5'-GTG AGT CCA GGA GAT GTT TCA and 5'-CTG TTG AGA TGA AAG GAG ACA) and *A3Z1 *exons 3–5 (primers 5'-CAT CTC AAC AGG GTT TGG ATC A and 5'-NNN NGT CGA CGT TTT GCT GAG TCT TGA G). The resulting PCR amplicon was digested with EcoRI and SalI and then ligated into a similarly cut pEGFP-N3 (Clontech).

### Rif^R ^DNA deamination assays

Cytosine deaminase activity of the artiodactyl A3 protein variants was measured by quantifying the accumulation of Rif^R ^mutants in *ung*-deficient *E. coli *(*e.g*., [42,56]). All A3 proteins were expressed from pTrc99A (AP Biotech), with the exception of cow A3Z1 and human A3A, which were expressed using pCR-BLUNT-II-TOPO (Invitrogen). Experiments were done a minimum of three times, in the presence or absence of IPTG as indicated.

### Fluorescence microscopy

To observe subcellular localization of A3 proteins, 5000 Hela cells were incubated for 24 h in Labtek chambered coverglasses (Nunc), transfected with 200 ng of the pEGFP-N3 based constructs and, after an additional 24 h visualized on a Zeiss Axiovert 200 microscope at 400× magnification. HsA3F, HsA3C, HsA3H, HsA3A, BtA3Z2-Z3 and SsA3Z2-Z3 fusion constructs were previously reported [19,42,79].

### Phylogenies and positive selection calculations

Z domain exons were used for all phylogenetic, positive selection and modelling studies. GARD showed no evidence for recombination breakpoints within the Z domain exons [81]. T_coffee version 5.31 was used for multiple sequence alignments [82]. PAL2NAL software was used to convert amino acid sequences to nucleotides [83]. JalView was used to remove insertions/deletions [84]. The dnaml program within the Phylip software package was used to generate a phylogenetic tree ([85]; an identical tree was obtained with MrBayes version 3.1 [86], except branch lengths differed slightly). Clustal W version 1.83.1 was also used for some individual domain comparisons [87].

Free ratio model positive selection studies were based on a phylogenetic tree generated through Bayesian inference using MrBayes version 3.1 [86]. Each tree was run for 250,000 generations with a burnin of 62,500 and standard default parameters. The PAML codeml program [88] was used to generate dN/dS ratios (ω values) for phylogenetic tree branches. ω values from the free ratio model using the F3 × 4 algorithm are shown in Additional File 6 (values from the F1 × 61 algorithm were similar and therefore not shown).

Positive selection was also evaluated in specific phylogenetic lineages using the NsSites model in the PAML codeml program (Table 1). Individual Z domain phylogenetic trees were generated as described above and used in these analyses. Z2 and Z3 comparisons were done for sheep, cow, pig, peccary and horse sequences, and Z1 comparisons for sheep, cow, horse and dog sequences (non-artiodactyl sequences were added for statistical significance; GenBank accession numbers are in Additional File 3). Models for neutral selection (M1 and M7) were compared to those for positive selection (M2 and M8). Likelihood ratio tests were performed to compare the null and positive selection scenarios.

### *A3 *gene expansion modelling

The aim was to infer the most likely histories of duplications and deletions that gave rise to the human *A3 *locus. Instead of considering each gene as an individual element, we subdivided it into its N-terminal and C-terminal Z-domains. Hence, the present-day human locus configuration was represented as follows: Z1-Z2-Z1-(Z2)_6_-Z1-Z3. The considered duplications are 'multiple tandem duplications' resulting from unequal crossing over [70]. In other words, a single duplication event can copy an arbitrary number of consecutive Z-domains, and place them in the same order next to the original ones. Similarly, an unequal crossing-over can remove an arbitrary number of adjacent domains and cause deletions.

Various algorithms have been proposed to infer evolutionary histories of tandemly arrayed gene families [71,89-91], but none of them involve both multiple tandem duplications and deletions. Consequently, we developed a brute force algorithm to enumerate all possible evolutionary scenarios involving a minimum number of duplications and deletions that can transform a particular locus configuration into another. Such an exhaustive algorithm has an exponential time complexity and it is impractical for analyzing large gene families. However, the limited size of the *A3 *locus and the classification of the Z domains into three distinct categories made it useful here (*e.g*., events 1 to 4 in Figure 7 & Additional File 7).

To infer the most recent evolutionary events (events 5 to 8 in Figure 7 & Additional File 7), we performed an analysis of the self-similarities within the human *A3 *locus. The DNA sequence (hg18, chr22:37682569-37830946) with identified interspersed repeats was downloaded from the RepeatMasker web site . A dot plot of this sequence with itself was obtained using Gepard [92] to identify pairs of regions with very high similarities. The three most significant were extracted and further aligned using Blastz [93] with default parameter to obtain the percentage of identity. These regions were used to infer and model the most recent evolutionary events, as described in the main text.

## Data deposition

The GenBank accession number for the sheep *A3 *genomic sequence is FJ042940. The GenBank accession numbers for the two pig *A3 *genomic sequences are FJ042938 and FJ042939. All *A3 *cDNA and EST sequences have also been deposited (see Additional File 3 for a full list of GenBank accession numbers).

## Abbreviations

A.A.: amino acid; A3: APOBEC3; A3A: APOBEC3B; A3B: APOBEC3C; A3C: APOBEC3C; A3DE: APOBEC3DE; A3F: APOBEC3F; A3G: APOBEC3G; A3H: APOBEC3H; GFP: Green Fluorescent Protein; PAML: Phylogenetic Analysis by Maximum Likelihood; MYA: Millions of Years Ago; Z: Zinc-coordinating motif

## Authors' contributions

RSL and RSH designed the studies, performed experiments, analyzed data and wrote the manuscript. SRJ and VA helped analyze the artiodactyl A3 genes and proteins, TPLS provided library samples and generated genomic DNA sequences, IH contributed cattle A3 gene sequences and functional data, KATS assisted with phylogenetic and computational studies, and ML, DB and NE generated the model for A3 evolution. All authors contributed to editing the manuscript.

## Supplementary Material

Additional file 1 **APOBEC3 Z domain conservation**. Web LOGO profiles depicting amino acid conservation within each mammalian Z domain. The multiple sequence alignments used to generate the phylogenic tree in Figure 1 were used to create consensus profiles for each of the indicated Z domains using Web LOGO [94]. Arrowheads below the amino acid profiles indicate residues that define each Z type (see the main text for additional details). Click here for file

Additional file 2 ***APOBEC3*****genomic locus comparisons**. Dotplot alignments of (*A*) the sheep and pig and (*B*) the sheep and cow *A3 *genomic sequences. Click here for file

Additional file 3 **Mammalian *A3 *and *AID *sequences**. A table summarizing all DNA sequences used in this study, including GenBank accession numbers. Click here for file

Additional file 4 ***APOBEC3*****promoter element conservation**. Predicted interferon-stimulating response elements (ISRE) in the promoter regions of the indicated *A3 *genes and known interferon-inducing genes *ISG54 *and *ISG15*. The ISRE sequences are shown relative to the translation initiation codon ATG. Identities to human sequences are shaded gray. Click here for file

Additional file 5 ***E. coli*****-based DNA cytosine deaminase activity data**. DNA cytosine deaminase activity of the pig A3Z2-Z3 and A3Z2 proteins in *E. coli*. Conditions and labels are identical to those used in Figure 4, except 10 independent cultures were grown under IPTG-induced conditions and analyzed. Click here for file

Additional file 6 **Evidence for positive selection in*****APOBEC3*****gene evolution**. Phylogenetic trees showing relative relationships and ω values for the indicated (*A*) Z1 domains, (*B*) Z2 domains, (*C*) Z3 domains and (*D*) the Z domain of *AID*. The phylogenetic trees were determined using MrBayes, and the ω values were calculated using the PAML free ratio model. ω values are shown in red adjacent to (or where space is non-permitting, to right of) each phylogenetic branch. Asterisks denote branches where the ω value was infinity (*i.e.*, dS was zero). The units for the scale bars are nucleotide changes per codon. The dotted line in panel (*C*) was used to provide more space to depict the human and non-human primate Z3 tree branches. See the main text and Methods for additional details. Click here for file

Additional file 7 **Proposed*****APOBEC3 *****gene diversification events during primatification**. An alternative representation of the 8-event model for the duplication and deletion history of the human *A3 *repertoire. Z1, Z2 and Z3 domains are colored green, orange and blue, respectively. The Z domain(s) involved in each event are shaded gray. Dark black and red lines mark duplications (one color for the original segment and one color for the duplicated segment), crosses designate deletions and light gray lines indicate no change. See the main text, Figure 7 and Methods for details. Click here for file

Additional file 8 **Primers used to identify expressed*****APOBEC3*****transcripts from cow, sheep and pig PBMCs**. A table summarizing the oligonucleotide primers used in this study. Click here for file
