# Supplementary material for: The artiodactyl APOBEC3 innate immune repertoire shows evidence for a multi-functional domain organization that existed in the ancestor of placental mammals
Source: BMC Mol Biol. 2008 Nov 18;9:104. doi: 10.1186/1471-2199-9-104 (PMC2612020; doi:10.1186/1471-2199-9-104)
Supplement: Additional file 7 — ProposedAPOBEC3 gene diversification events during primatification. An alternative representation of the 8-event model for the duplication and deletion history of the human A3 repertoire. Z1, Z2 and Z3 domains are colored green, orange and blue, respectively. The Z domain(s) involved in each event are shaded gray. Dark black and red lines mark duplications (one color for the original segment and one color for the duplicated segment), crosses designate deletions and light gray lines indicate no change. See the main text, Figure 7 and Methods for details. [file 1471-2199-9-104-S7.doc]

**Table S1. Mammalian *A3* and *AID* sequences**

| **Species** | **Common name** | **Protein** | **Accession number** | **Journal** |
| --- | --- | --- | --- | --- |
| *Bos taurus* | Cow | A3Z1 | EU864534 | This study |
| *Bos taurus* | Cow | A3Z2-Z3 | DQ974646 | Jónsson *et al.,* 2006 |
| *Bos taurus* | Cow | A3Z2 | EU864535 | This study |
| *Bos taurus* | Cow | A3Z3 | EU864536 | This study |
| *Bos taurus* | Cow | AID | NM_001038682 | Verma and Aitken, 2005 |
| *Ovis aries* | Sheep | A3Z1 | EU864541 | This study |
| *Ovis aries* | Sheep | A3Z2-Z3 | DQ974646 | Jónsson *et al.,* 2006 |
| *Ovis aries* | Sheep | A3Z2 | EU864542 | This study |
| *Ovis aries* | Sheep | A3Z3 | EU864543 | This study |
| *Ovis aries* | Sheep | AID | EE793762 | This study |
| *Sus scrofa* | Pig | A3Z2-Z3 | DQ974646 | Jónsson *et al.,* 2006 |
| *Sus scrofa* | Pig | A3Z2 | EU864539 | This study |
| *Sus scrofa* | Pig | A3Z3 | EU864540 | This study |
| *Sus scrofa* | Pig | AID | BP157753 | This study |
| *Tayssu tajacu* | Peccary | A3Z2-Z3 | EU864537 | This study |
| *Tayssu tajacu* | Peccary | AID | EU864538 | This study |
| *Equus caballus* | Horse | A3Z1 | XM_001499871 | Genome project ID: 19129 |
| *Equus caballus* | Horse | A3Z2 | XM_001501833 | Genome project ID: 19129 |
| *Equus caballus* | Horse | A3Z3 | XM_001501833 | Genome project ID: 19129 |
| *Felis catus* | Cat | A3Z2b-Z3 | EF173021 | Munk *et al*., 2007 |
| *Canis lupus* | Dog | A3Z1 | XM_847690 | Genome project ID: 12384 |
| *Canis lupus* | Dog | A3Z2 | AACN010393938 | Kirkness *et al*., 2003 |
| *Canis lupus* | Dog | A3Z3 | XM_538369 | Genome project ID: 12384 |
| *Canis lupus* | Dog | AID | NM_001003380 | Ohmori *et al.,* 2004 |
| *Mus musculus* | Mouse | A3Z2-Z3 | NM_030255 | Many; LaRue *et al*., 2008 |
| *Mus musculus* | Mouse | AID | NM_009645 | Many; LaRue *et al*., 2008 |
| *Rattus norvegicus* | Rat | A3Z2-Z3 | NM_001033703 | Strausberg *et al.,* 2002 |
| *Rattus norvegicus* | Rat | AID | XM_001060382 | Twigger *et al*., 2007 |
| *Homo sapiens* | Human | A3A (A3Z1a) | NM_145699 | Many; LaRue *et al*., 2008 |
| *Homo sapiens* | Human | A3B (A3Z2a-Z1b) | NM_004900 | Many; LaRue *et al*., 2008 |
| *Homo sapiens* | Human | A3C (A3Z2b) | NM_014508 | Many; LaRue *et al*., 2008 |
| *Homo sapiens* | Human | A3DE (A3Z2c-Z2d) | NM_152426 | Many; LaRue *et al*., 2008 |
| *Homo sapiens* | Human | A3F (A3Z2e-Z2f) | NM_145298 | Many; LaRue *et al*., 2008 |
| *Homo sapiens* | Human | A3G (A3Z2g-Z1c) | NM_021822 | Many; LaRue *et al*., 2008 |
| *Homo sapiens* | Human | A3H (A3Z3) | NM_181773 | Many; LaRue *et al*., 2008 |
| *Homo sapiens* | Human | AID | NM_020661 | Many; LaRue *et al*., 2008 |
| *Pan troglodytes* | Chimp | A3H | DQ408607 | OhAinle *et al*., 2006 |
| *Pan troglodytes* | Chimp | AID | NM_001071809 | Zhou *et al.,* 2005 |
| *Macaca mulatta* | Macaque | A3H | XM_001096739 | Genome project ID: 16397 |
| *Macaca mulatta* | Macaque | AID | XM_001113641 | Genome project ID: 16397 |
| *Hylobates syndactylus* | Gibbon | A3H | DQ408608 | OhAinle *et al.,* 2006 |
| *Pongo pygmaeus* | Orangutan | A3H | DQ408610 | OhAinle *et al.,* 2006 |
| *Gorilla gorilla* | Gorilla | A3H | DQ408609 | OhAinle *et al.,* 2006 |
| *Pan paniscus* | Bonobo | A3H | DQ408606 | OhAinle *et al.,* 2006 |
| *Miopithecus talapoin* | Talapoin | A3H | DQ408613 | OhAinle *et al.,* 2006 |
| *Papio anubis* | Baboon | A3H | DQ408605 | OhAinle *et al.,* 2006 |
| *Cercocebus atys* | Sooty mangabey | A3H | DQ408611 | OhAinle *et al*., 2006 |
| *Saguinus labiatus* | Tamarin | A3H | DQ408614 | OhAinle *et al*., 2006 |
| *Ateles belzebuth* | Spider | A3H | DQ408612 | OhAinle *et al*., 2006 |
| *Lagothrix lagotricha* | Woolly monkey | A3H | DQ408615 | OhAinle *et al*., 2006 |
