# Supplementary material for: The artiodactyl APOBEC3 innate immune repertoire shows evidence for a multi-functional domain organization that existed in the ancestor of placental mammals
Source: BMC Mol Biol. 2008 Nov 18;9:104. doi: 10.1186/1471-2199-9-104 (PMC2612020; doi:10.1186/1471-2199-9-104)
Supplement: Additional file 8 — Primers used to identify expressedAPOBEC3transcripts from cow, sheep and pig PBMCs. A table summarizing the oligonucleotide primers used in this study. [file 1471-2199-9-104-S8.doc]

**Table S2.** Primers used to identify expressed *APOBEC3* transcripts from cow, sheep and pig PBMCs.

|  | **Outer**  **5’ RACE** | **Inner**  **5’ RACE** | **Outer**  **3’ RACE** | **Inner**  **3’ RACE** |
| --- | --- | --- | --- | --- |
| Cow A3Z1 | ACTCTGATGGCACCCAAAAC | GCCTCTCCATCTCGTAGCAC | TTTGGATCAACCGGAGAAAC | TCCTGAAGGAGAACCACCAC |
| Cow A3Z2-Z3 | ATGCTCAGCGTCACATTCTG | GGAGGCACGTGAGTGGTATT | ACCAGCTGAAGCAGCGTAAT | GCATAAGACGAAGGCTCCAG |
| Cow A3Z2 | Same as cow A3Z2-Z3 | Same as cow A3Z2-Z3 | CAGAATGTGACGCTGAGCAT | GGAGCCCATGTGGACATTAT |
| Cow A3Z3 | n.a. | n.a. | Same as cow A3Z2-Z3 | Same as cow A3Z2-Z3 |
| Sheep A3Z1 | n.a. | n.a. | TCCGTTCTTGGAATCTGGAC | GAAGGAGAACCGCCACATAA |
| Sheep A3Z2-Z3 | TCAGCGTCACATTCTGGTACA | AGTCCCAGCATAGACCTGGTT | AACCAGGTCTATGCTGGGACT | CTGGGGATGTACCAGAATGTG |
| Sheep A3Z2 | Same as sheep A3Z2-Z3 | Same as sheep A3Z2-Z3 | Same as sheep A3Z2-Z3 | Same as sheep A3Z2-Z3 |
| Sheep A3Z3 | n.a. | n.a. | Same as sheep A3Z2-Z3 | Same as sheep A3Z2-Z3 |
| Pig A3Z2-Z3 | n.a. | n.a. | CCAAGGAGCTGGTTGATTTC | CTGGAGCAATACAGCGAGAG |
| Pig A3Z2 | n.a. | n.a. | ACGTCACCTGGTTCATCTCC | ACTGCTGGAACAACTTCGTG |
| Pig A3Z3 | n.a. | n.a. | Same as pig A3Z2-Z3 | Same as pig A3Z2-Z3 |

n.a. = not applicable
